# Relationship between shear elastic modulus and passive muscle force in human hamstring muscles using a Thiel soft-embalmed cadaver

**DOI:** 10.1007/s10396-023-01317-8

**Published:** 2023-05-11

**Authors:** Gakuto Nakao, Taiki Kodesho, Takuya Kato, Yu Yokoyama, Yuhei Saito, Yuki Ohsaki, Kota Watanabe, Masaki Katayose, Keigo Taniguchi

**Affiliations:** 1 Graduate School of Health Sciences, Sapporo Medical University, Sapporo, Japan; 2 Professional Post-Secondary Course (Physical Therapist), Sapporo Medical Technology, Welfare and Dentistry Professional Training College of Nishino Gakuen School Foundation, Sapporo, Japan; 3 Department of Rehabilitation, Hitsujigaoka Hospital, Sapporo, Japan; 4 Department of Rehabilitation, Heiseikai Hospital, Sapporo, Japan; 5 First Division of Anatomy, School of Medicine, Sapporo Medical University, Sapporo, Japan; 6 Department of Physical Therapy, School of Health Sciences, Sapporo Medical University, South-1, West-17, Chuo-Ku, Sapporo, Hokkaido 060-8556 Japan

**Keywords:** Shear wave elastography, Passive muscle force, Muscle architecture, Hamstrings

## Abstract

**Purpose:**

Assessing muscle flexibility and architecture is important for hamstring strain injury (HSI) prevention. We investigated the relationship between shear modulus and passive force in hamstring muscles at different sites and the effect of muscle architecture on the slope of the shear modulus–passive force using shear wave elastography (SWE).

**Methods:**

The biceps femoris long head (BFlh), semitendinosus (ST), and semimembranosus (SM) muscles were dissected from nine Thiel-embalmed cadavers and fixed to a custom-made mechanical testing machine. Calibrated weights (0−1800 g) were applied gradually in 150-g increments. The shear modulus and anatomical cross-sectional area (ACSA) were measured at proximal, central, and distal points using SWE. The muscle mass and length were measured before the loading test. The shear modulus–passive load relationship of each tested muscle region was analyzed by fitting a least-squares regression line. The increase in shear modulus slope per unit load was calculated and compared between the muscles before and after normalization by the muscle mass, length, and ACSA.

**Results:**

The shear modulus and passive force for all hamstring muscles in each region showed a statistically significant linear correlation. Furthermore, the increase in shear modulus slope was greater for BFlh and ST than for SM (P < 0.05), but after normalization by the muscle length and ACSA, there were no significant differences among the muscles.

**Conclusion:**

The local mechanical properties of individual hamstring muscles can be indirectly estimated using SWE, and the slope of increase in shear modulus reflects characteristics of the muscle architecture.

## Introduction

The incidence and recurrence of hamstring strain injury (HSI) are very high in several sports [1]. HSI is caused by rapid, extensive eccentric contractions or overstretching of the hamstring muscle group, resulting in high mechanical stress [2]. Two specific types of HSI based on the injury mechanism have been described: (1) sprint-type injuries that occur during high-speed running and mainly involve the biceps femoris long head (BFlh) [3] and (2) stretch-type injuries that occur during extensive hamstring lengthening and often involve the semimembranosus (SM) [3]. Both injuries frequently affect the proximal muscle–tendon junction [4] and have different post-injury effects (e.g., sprint-type injuries have a shorter recovery time than stretch-type injuries). The incidence of HSI in the semitendinosus (ST) has been reported to be lower than that in the BFlh and SM, but with more injuries at the proximal site, and with regional differences in HSI as in the BFlh and SM [5]. Therefore, these previous reports have indicated that some muscles and sites are more susceptible to HSI, and detailed assessment of mechanical stress among muscles and by the site may provide important information regarding injury prevention.

Previous studies have indicated that the etiology of HSI is multifactorial, related to decreased muscle flexibility and changes in muscle architecture [6–10]. Witvrouw et al. showed that players with HSI had significantly lower hamstring flexibility before the injury compared with those who were not injured [8]. In addition, a prospective study of elite soccer players by Timmins et al. found a 4.1-fold increased risk of HSI with a shorter BFlh length [9]. It has been reported that individuals with a history of unilateral HSI have shorter BFlh fascicles in the previously injured limb than in the contralateral uninjured limb [10]. This suggests that shorter BFlh fascicles may be associated with an increased risk of HSI, although it can be speculated that addressing these issues will likely reduce the risk of HSI. Therefore, assessing muscle flexibility and architecture is very important for HSI prevention. Ultrasonic shear wave elastography (SWE), which can measure local tissue shear modulus, has recently been used to quantify the passive material properties of individual muscles [11]. The relationship between passive muscle force–shear modulus has been demonstrated in muscles, such as the tibialis anterior in chickens [12], lower leg muscles in swine [13], and the adductor longus and rectus femoris muscles in human cadavers [14, 15]. Thus, a strong linear relationship between muscle shear modulus and passive muscle force, obtained using SWE, indicates that the mechanical stresses associated with individual muscle stretching can be estimated [12–15]. However, this relationship in individual hamstring muscles has not been established. In previous studies that measured the mechanical properties of each hamstring muscle using SWE, the hamstring muscles with the greatest shear modulus in the extended position differed among human studies [16–18]. Thus, it remains unclear whether the change in the shear modulus of the hamstring muscles reflects the passive force with muscle elongation.

Furthermore, the slope of the shear modulus–passive force may be affected by muscle architecture [12–14]. Kato et al. and Koo et al. reported that the slope of increase in shear modulus was correlated with the muscle mass and anatomical cross-sectional area (ACSA) [12, 14]. In contrast, Liu et al. reported no significant difference in the slope of change in shear modulus among muscles when normalized by muscle mass, even for muscles with different muscle mass [13]. Considering that the slope of the shear modulus–passive force has been shown to be influenced by the muscle mass and ACSA in vitro, individual hamstring muscles with differences in structural characteristics, such as muscle mass, muscle length, and ACSA, would likewise exhibit a slope that is dependent on muscle architecture [19, 20].

Therefore, this study aimed to determine the relationship between the shear modulus and passive muscle force of individual hamstring muscles at different sites using Thiel soft-embalmed cadavers and investigate the effect of muscle architecture in each hamstring muscle on the slope of the shear modulus–passive force.

## Materials and methods

### Preparation of human cadavers

The BFlh, ST, and SM were dissected from nine Thiel soft-embalmed cadavers (mean age at death: 86 years, range: 78–96 years; mean height at death: 164 cm, range: 150–164 cm; mean weight at death: 63 kg, range: 52–69 kg). The Thiel method is an ingenious technique that uses preservatives other than formaldehyde and allows tissues, especially muscles, to retain most of their prenatal texture and tone [21]. Previous studies have reported that the mechanical properties of specimens obtained using the Thiel method are similar to those of biological skeletal muscles and tendons [22, 23]. Beger et al. reported that Young's modulus, which represents the longitudinal modulus of elasticity under tensile loads, was not significantly different between the Thiel-embalmed and frozen/thawed gastrocnemius muscles of rats [22]. In addition, Hohmanna et al. reported no difference in a tendon between the mechanical properties of fresh frozen specimens and Thiel method specimens [23]. Therefore, the mechanical properties of the Thiel method-fixed hamstring muscle specimens used in our study are expected to be similar to those of biological skeletal muscles.

The common tendon of BFlh and ST was dissected from the inferomedial part of the ischial tuberosity at the origin. The attachments of BFlh and ST were dissected from the head of the fibula and the medial surface of the tibia, respectively. SM was dissected at the superolateral part of the ischial tuberosity and the posterior surface of the medial tibial condyle. During the experiments, the room temperature and humidity were maintained at 22 °C and 40%, respectively, so that the histological properties of the specimens were not affected by changes in temperature or humidity. The study protocol was approved by the appropriate ethics committee (approval number: 4-1-70).

### Experimental setup

**Fig. 1:**
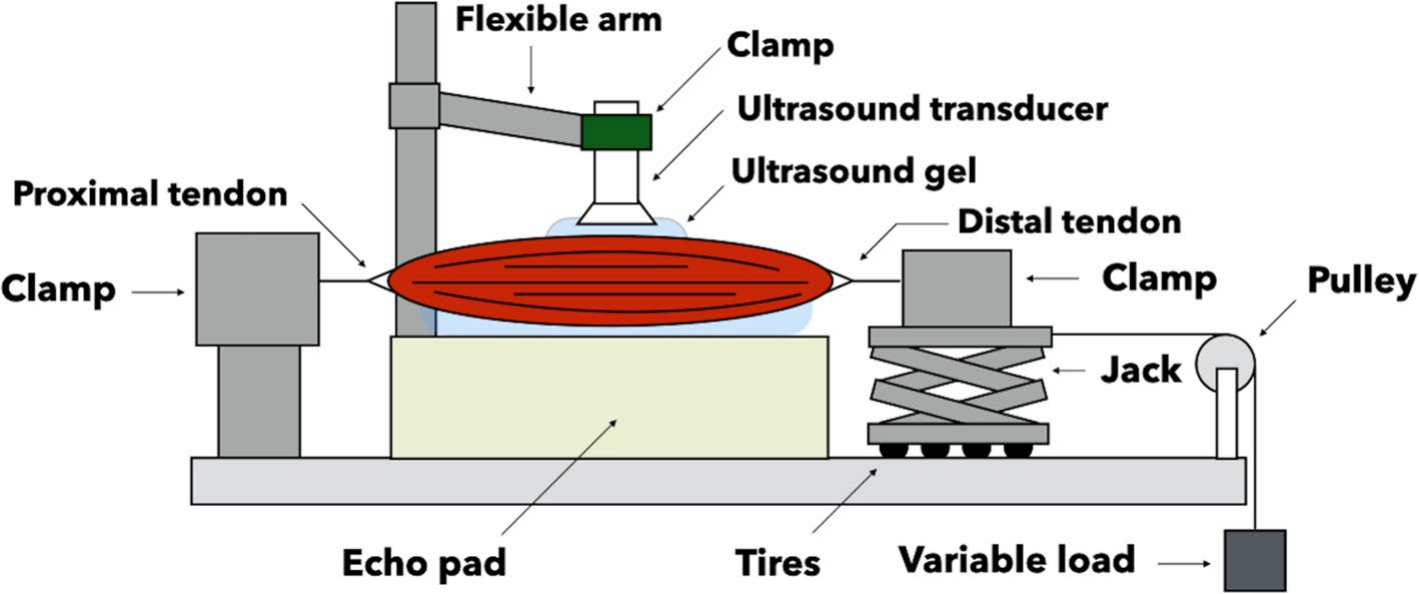
Schematic of the experimental setup used to apply passive force to the distal end of the muscle. Lubricating gel was applied between the muscle and the echo pad to reduce friction when pulling on the muscle

The experimental setup was prepared based on previous studies [14, 15, 24]. In order to apply passive tension to individual hamstring muscles, specimens were fixed to a custom-built device (Uchida Systems Co., Ltd., Tokyo, Japan) (Fig. 1). The proximal and distal tendons of the muscle were each immobilized with different clamps. A height-adjustable jack on very smooth tires was attached under the base of the clamp, immobilizing the distal tendon and rendering the long axis of the muscle horizontal to the floor. The base of the clamp immobilizing the distal tendon was connected to a cable, which provided a passive load to the muscle via a pulley. With respect to the load setting, the protocol in the previous experiment used 400 g as the maximum load for the chicken gastrocnemius muscle [12]. The muscle mass in the previous study was approximately 22 g. In contrast, the muscle mass in this study averaged 100 g, and the muscle mass in this experiment was approximately 4.5 times greater than that in the previous study. Therefore, the passive load was increased in steps of 150-g increments from 0 to 1800 g, which corresponds to 4.5 times the load in the previous study. To minimize tissue creep and hysteresis effects caused by prolonged passive tension, the shear modulus measurement time at each passive tension was limited to ≤ 10 s. The load was removed immediately after measurement, and the next load was applied after 30 s. This passive loading experiment was performed twice at 3-min intervals between experiments.

### Measurement of muscle architecture and shear modulus

**Fig. 2:**
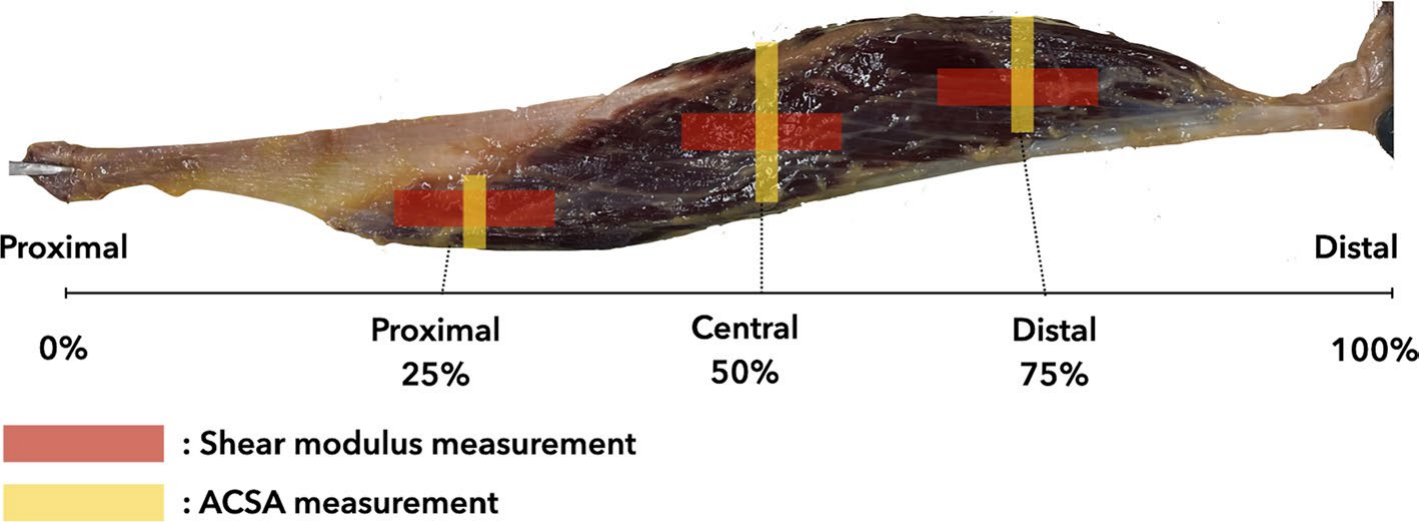
Shear modulus and anatomical cross-sectional area (ACSA) measurement area in the biceps femoris long head muscle

Before the passive load experiment, the muscle length, muscle mass, and ACSA were measured. The muscle length was measured in 1-mm increments using the distance between the proximal and distal ends of the separated tendons. The muscle mass was measured in 1-g increments using a digital scale (SH-5000; A&D Co., Ltd., Tokyo, Japan). ACSA was measured at the proximal third (25%), central (50%), and distal third (75%) regions at a distance from the proximal to the distal end of the separated tendon of the muscle under an extended field of view using B-mode ultrasonography (Aixplorer Ver. 12, MSK mode; Hologic, Marlborough, MA, USA) with a 4–15-MHz linear ultrasound transducer (Fig. 2).

Muscle shear modulus was measured using SWE with a linear ultrasound transducer (4–15 MHz) (Aixplorer Ver. 12, MSK mode; Hologic). Young’s modulus ranged from 0 to 300 or 400 kPa. Blue and red on Young’s modulus (in kilopascals) color scale correspond to the lowest and highest values, respectively. After the specimen was set into the device, a 30-mm-thick ultrasound echo pad (Echo PAD; Yasojima Proceed Co., Ltd., Amagasaki, Japan) was set under the muscle. The transducer was placed on the muscle. Ultrasound gel was applied between the echo pad and muscle and between the echo pad and transducer. A flexible arm was used to position and hold the transducer to ensure that the same measurement site could be imaged at the same orientation throughout the passive loading experiment. For the shear modulus and ACSA measurement sites, measurements were performed in the proximal, central, and distal regions (Fig. 2). The shear modulus measurement site was determined using an ultrasonic B-mode longitudinal image including a clear fascicle of each region of the individual muscles. The shear modulus was measured twice for each load.

### Data analysis

**Fig. 3:**
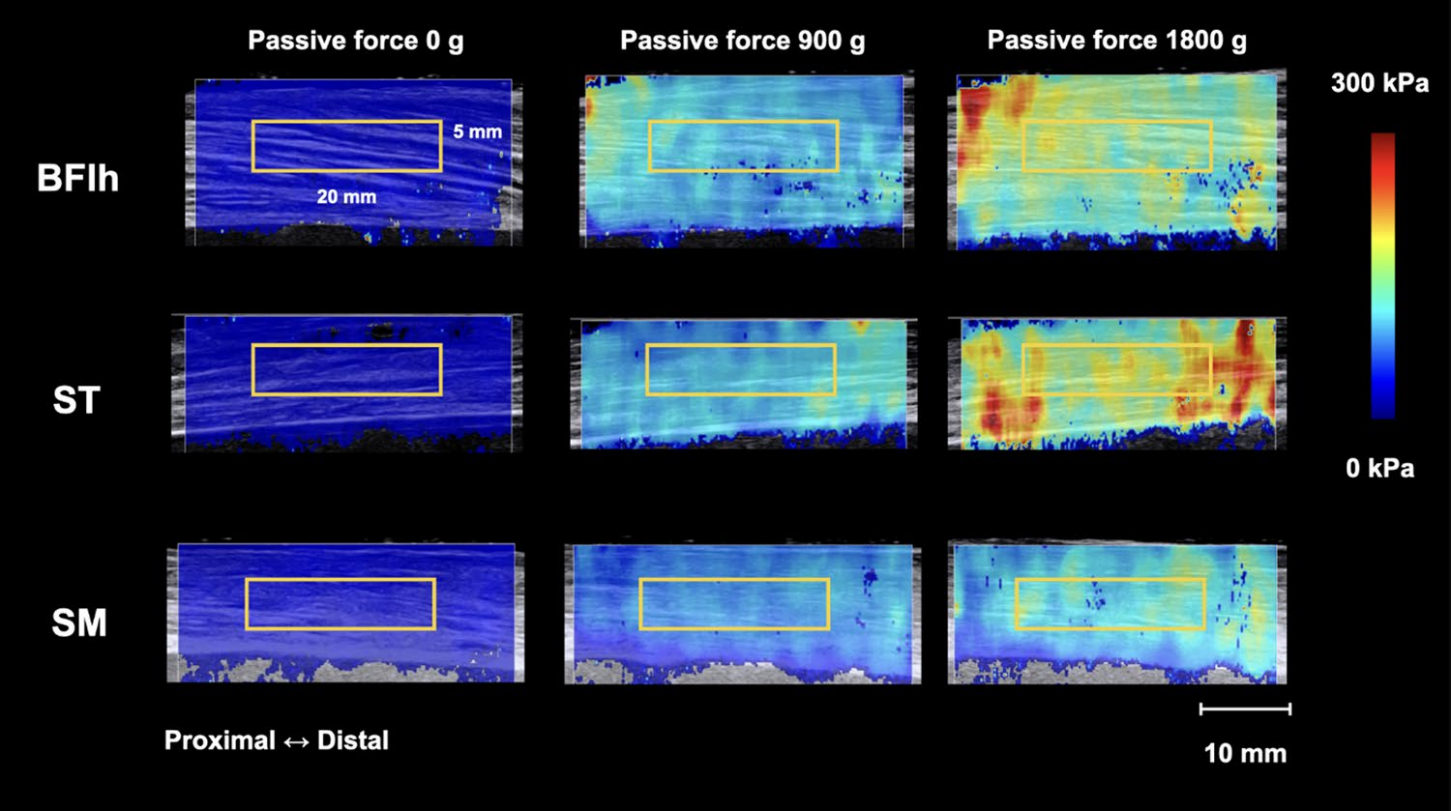
Typical examples of the shear wave elastography (SWE) image when 0-g, 900-g, and 1800-g loads were applied. The colored region represents the shear modulus map with the scale to the right of the figure. Rectangle is the region of interest for the determination of muscle shear modulus

The B-mode ultrasound image with a frame rate of 44 Hz captured each muscle region in the transverse section. Then, the ACSA was calculated using the measurement tool of B-mode ultrasonography to measure the outline of the fascial muscle line by moving the marks displayed on the screen with a trackball. For shear modulus measurement, the shear modulus images in the JPEG format were exported and analyzed using custom analysis software (S-14133 Ver. 1.2; Takei Scientific Instrument Co., Ltd., Niigata, Japan) [14]. With this software, the region of interest (ROI) can be arbitrary in size and shape and can be placed anywhere on the shear modulus image. The elastic modulus calculation was based on the color map scale. A rectangular ROI (width, 20 mm; height, 5 mm) was set on the color map to include many muscle fascicles in accordance with the previous study (Fig. 3) [14]. The center of the ROI height was aligned with the center of the muscle thickness. Using the SWE software, Young's modulus was quantified in kilopascals based on the shear wave propagation speed (c). For each ROI, Young’s modulus (E) was deduced from E = 3ρc^2^, where ρ (density) was assumed to be constant (1000 kg/m^3^) in human soft tissues. This SWE software calculates Young's modulus based on the assumption that biological tissue is an isotropic material, while the muscle is not [25]. Therefore, we analyzed the shear modulus by dividing Young's modulus by 3. For each image under load conditions, the average shear modulus within a rectangular ROI was calculated. The mean of two images was regarded as the shear modulus of the tested location at that load. After calculating the average shear modulus, the slope of increase in shear modulus per unit load was calculated. The slope of increase in shear modulus per unit load, s, was calculated as [s = (G_1800_ − G_0_)/F, the shear modulus of the muscle due to 1800 g of passive force; (G_1800_), the shear modulus of the muscle at its slack length; (G_0_), and the passive force; (F), 1800 g]. The slope of the increase in shear modulus per unit load was normalized by multiplying (1) the muscle mass and (2) the pseudo-muscle volume calculated by multiplying the average ACSA of the three measured sites by the muscle length.

### Statistical analysis

All statistical analyses were performed using SPSS Statistics Ver. 28.0 (IBM Corp., Armonk, NY, USA). Intraclass correlation coefficient (ICC) estimates were calculated based on mean ratings (k = 2), absolute agreement, and a two-way mixed-effect model to evaluate the test–retest reliability of the ACSA and SWE measurements [26]. The shear modulus–passive muscle force relationship of each tested muscle region was analyzed by fitting a least-squares regression line. Two-way repeated-measures analysis of variance (ANOVA), using the factors of muscles (BFlh, ST, and SM) and sites (proximal, central, and distal), was used to analyze interaction effects for the slope of increase in shear modulus. A one-way repeated-measures ANOVA was used to determine differences in muscle length, muscle mass, and ACSA among muscle groups. When a significant main effect was observed, a post-hoc analysis with Bonferroni correction was performed where appropriate. The significance level was set at 5% for all tests.

## Results

### Reproducibility of the ACSA and SWE measurements

The test–retest reliability results for ACSA and shear modulus are presented in Table 1. The test–retest reliability was excellent for all muscles and sites.

**Table 1:** Intraclass correlation coefficient for anatomical cross-sectional area (ACSA) and shear modulus in each hamstring muscle at each site for all tested muscles (n = 9)

	Proximal	95%CI	Central	95%CI	Distal	95%CI	Proximal	95%CI	Central	95%CI	Distal	95%CI
BFlh	0.997	(0.991–0.998)	0.994	(0.990–0.996)	0.996	(0.993–0.997)	0.994	(0.989–0.998)	0.987	(0.982–0.993)	0.995	(0.991–0.998)
ST	0.995	(0.990–0.998)	0.994	(0.989–0.997)	0.995	(0.990–0.999)	0.994	(0.988–0.997)	0.993	(0.991–0.998)	0.993	(0.988–0.997)
SM	0.998	(0.994–0.999)	0.995	(0.991–0.998)	0.996	(0.991–0.997)	0.996	(0.992–0.998)	0.996	(0.989–0.998)	0.994	(0.992–0.996)

*ACSA* anatomical cross-sectional area, *CI* confidence interval, *BFlh* biceps femoris long head, *ST* semitendinosus, *SM* semimembranosus

### Relationship between shear modulus and passive muscle force

**Fig. 4:**
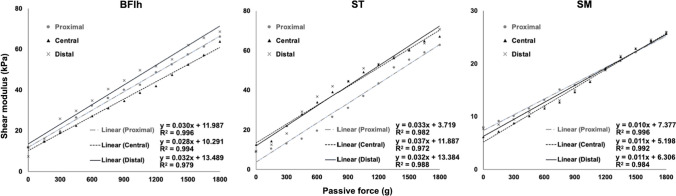
Typical example of a shear modulus–load plot of the biceps femoris long head (BFlh), semitendinosus (ST), and semimembranosus (SM) at each site

Typical examples of elasticity images are presented in Fig. 4. There was a linear correlation between the shear modulus and passive muscle force for all hamstring muscles in each region (P < 0.01) (Table 2).

**Table 2:** Mean of regression coefficients of shear modulus in individual hamstring muscles in all regions

	Proximal	Central	Distal
BFlh	0.984 (± 0.015; range, 0.968–0.997)	0.984 (± 0.010; range, 0.973–0.996)	0.975 (± 0.027; range, 0.909–0.996)
ST	0.973 (± 0.029; range, 0.964–0.997)	0.965 (± 0.027; range, 0.935–0.997)	0.967 (± 0.040; range, 0.868–0.998)
SM	0.976 (± 0.038; range, 0.868–0.995)	0.977 (± 0.019; range, 0.935–0.997)	0.978 (± 0.017; range, 0.939–0.996)

*BFlh* biceps femoris long head, *ST* semitendinosus, *SM* semimembranosus

### Differences in muscle architecture among hamstring muscles

The mean muscle length, muscle mass, and ACSA of individual hamstring muscles at each site are summarized in Table 3. For muscle length, one-way ANOVA indicated a significant main effect of the muscle group (F = 6.774, P < 0.01). Bonferroni's post hoc test indicated that the muscle length of ST was significantly longer than that of SM. For muscle mass, one-way ANOVA indicated a significant main effect of the muscle group (F = 14.832, P < 0.01). Bonferroni's post hoc test indicated that BFlh and SM muscle mass was significantly greater than that of ST. For ACSA, one-way ANOVA indicated a significant main effect of the muscle group (F = 5.000, P = 0.02). Bonferroni's post hoc test indicated that the ACSA of SM was significantly greater than that of ST.

**Table 3:** Mean and SD of muscle length, muscle mass, and anatomical cross-sectional area (ACSA) of all tested muscles (n = 9)

	Length (cm)	Mass (g)	ACSA (cm^2^)			
			Proximal	Central	Distal	Average
BFlh	39.6 ± 5.3	104.7 ± 28.6**	3.0 ± 1.1	4.2 ± 1.4	2.4 ± 1.1	3.2 ± 1.1
ST	43.1 ± 4.9*	80.3 ± 29.0	2.7 ± 1.0	3.1 ± 1.5	2.0 ± 0.9	2.6 ± 0.9
SM	38.3 ± 4.9	118.3 ± 38.0**	3.0 ± 1.8	5.2 ± 2.3	3.9 ± 2.0	4.0 ± 1.7^†^

*ACSA* anatomical cross-sectional area, *BFlh* biceps femoris long head, *ST* semitendinosus, *SM* semimembranosus

^*^p < 0.05: Significantly longer than semimembranosus muscle

^**^p < 0.05: Significantly greater than semitendinosus muscle

^†^p < 0.05: Significantly greater than semitendinosus muscle

### Comparison of the slope of increase in shear modulus among the hamstring muscles

**Fig. 5:**
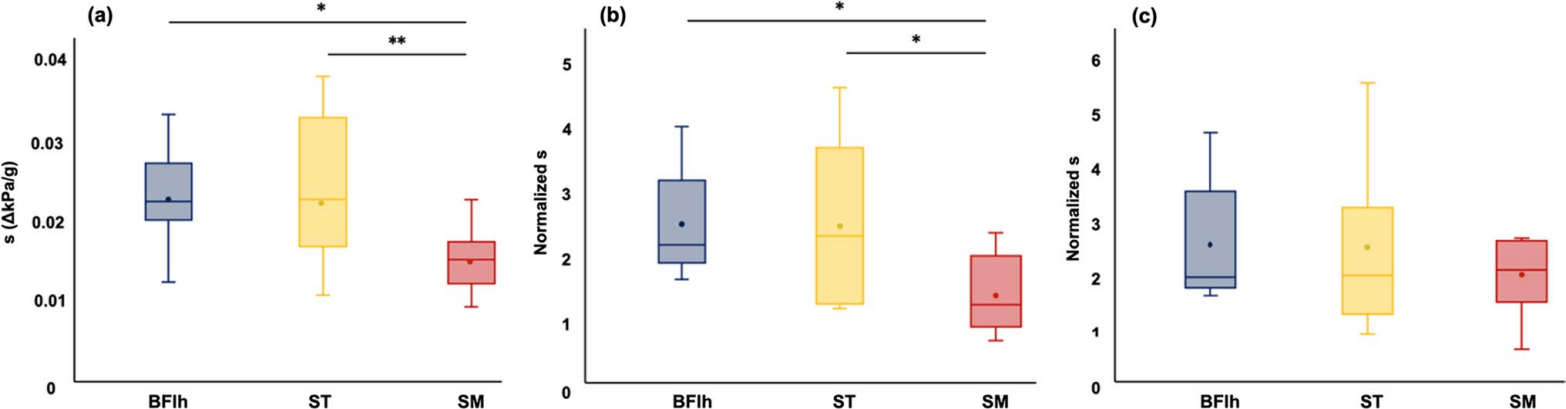
Comparison of the slope of increase in the shear modulus of individual hamstring muscles: **a** before normalization in muscle architecture, **b** after normalization in muscle mass, **c** after normalization in muscle length and ACSA. For each box, the interior line in bold shows the median, the small circle in the box is the mean value, and the edges of the box are estimates of the first and third quartiles. *BFlh* biceps femoris long head, *ST* semitendinosus, *SM* semimembranosus. *: P < 0.05: Significantly greater than the semimembranosus muscle. **: P < 0.01: Significantly greater than the semimembranosus muscle

The slope of increase in shear modulus two-way ANOVA revealed no significant interaction between muscles and sites (F = 1.020, P = 0.412). The main effect was observed for muscle (F = 8.265, P = 0.003) but not for sites (F = 1.077, P = 0.364). Post hoc test results showed that the increase in shear modulus slope was greater for the BFlh and ST than for the SM (P < 0.05), as shown in Fig. 5a. The slope of increase in shear modulus after normalization by muscle mass remained different among muscles as before normalization (P < 0.05) (Fig. 5b). However, after normalization by muscle length and ACSA, there were no significant differences among muscles in the slope of increase in shear modulus (P = 0.314) (Fig. 5c).

## Discussion

The main results of this study were as follows: (1) a strong correlation between shear modulus and passive muscle force was observed in individual hamstring muscles at each site, and (2) the slope of increase in shear modulus was significantly greater for the BFlh and ST than for the SM. However, after normalization by muscle length and ACSA, there were no significant differences among the muscles in the slope of increase in shear modulus. Previous studies on the relationship between muscle shear modulus and passive muscle force have been reported for lower limb muscles in animals, such as chickens [12] and swine [13], and in the adductor longus [14] and rectus femoris [15] muscles in human cadavers; however, no studies have examined this relationship in individual hamstrings of human cadavers. Furthermore, using Thiel soft-embalmed cadavers, no studies have compared the slope of increase in shear modulus of individual hamstring muscles normalized by muscle architecture. To the best of our knowledge, this is the first study to investigate the mechanical properties of individual hamstring muscles in different regions and compare the slope of increase in shear modulus normalized by muscle architecture in individual hamstrings using Thiel soft-embalmed cadavers.

As for the reproducibility of the shear modulus of individual hamstring muscles, the ICC was greater than 0.972 (0.972–0.998) for all measurement regions in this study (Table 1). Our results were consistent with those of previous studies [12–15] and were, thus, considered reliable. Moreover, a strong correlation was observed between the passive muscle force and shear modulus in individual hamstring muscles in all regions (Table 2). These results indicate that shear modulus accounts for more than 96% of the change in passive muscle force. Previous studies have demonstrated the relationship between passive muscle force and shear modulus in animal and human cadaver muscles [12–15]; a similar relationship was demonstrated in the hamstring muscles. The findings demonstrate that in each hamstring muscle's proximal, central, and distal regions, the shear modulus enables a highly accurate estimation of changes in the passive force.

The present results revealed that while differences remained in the slope of the increase in shear modulus among muscles when normalized by muscle mass, after normalization by muscle length and ACSA, there were no significant differences among muscles in the slope of the increase in shear modulus. This suggests that passive force generation is more closely related to muscle length and ACSA than to muscle mass in studying hamstring muscles in human cadavers. The slope changes of shear modulus normalized by the pseudo-muscle volume, calculated by multiplying the average ACSA and muscle length, can be explained by differences in the hamstring muscle architecture. The pseudo-volume of each hamstring muscle calculated in this study was the largest in SM and the smallest in ST (mean age of cadaver, 85.6 years; SM, 153.2 cm^3^; BF, 126.72 cm^3^; ST, 112.06 cm^3^). In a previous study using human cadavers, Storey et al. also reported that the volume of each hamstring muscle was the largest in SM and the smallest in ST (mean age of cadaver, 74.6 years; SM, 104.6 cm^3^; BF, 75.8 cm^3^; ST, 62.2 cm^3^) [27], and the same results were obtained by Takeda et al. (mean age of cadaver, 82.6 years; SM, 71.3 cm^3^; BF, 57.2 cm^3^; ST, 50.6 cm^3^) [28]. Therefore, the muscle volume calculated in this study may reflect the morphological characteristics of each hamstring muscle. Among the hamstring muscles, the BFlh and SM are characterized by a larger physiological cross-sectional area (PCSA) and shorter muscle fascicle length. In contrast, ST is characterized by a smaller PCSA and longer muscle fascicle length [19]. Furthermore, SM has a larger PCSA and shorter muscle fascicle length than BFlh [19]. Considering that the stress is the tensile force divided by the cross-sectional area, muscles with a large cross-sectional area may have less mechanical stress. When the same load was applied to individual muscles, as in this experiment, the SM with a smaller slope of increase in shear modulus could have taken advantage of the architectural properties of individual muscles, such as muscle length and ACSA, in response to the passive force. Further research, including examination of other muscle architectural features, is needed to investigate the exact mechanisms underlying the differences in shear modulus among hamstring muscles.

HSI can occur during various functional activities, but the most common injury mechanism is sprinting-related increased mechanical stress. The BFlh is exposed to higher tensile forces in the late swing phase of sprinting [29], which increases the risk of HSI. In previous studies that used SWE to measure muscle elasticity in each hamstring muscle during passive hip and knee motion [16–18], it was unclear whether the change in the shear modulus of the hamstring muscles reflected the passive force with muscle elongation. Based on the results from this study, it may be possible to estimate the change in the passive force of each hamstring muscle during passive hip and knee motion by quantifying the shear modulus of the hamstring muscles in living humans using SWE. The change in the passive force of the hamstring muscle during passive hip and knee motion may be useful information that can elucidate the mechanism of HSI. Moreover, this could help in the development of effective stretching exercises for the hamstring muscles.

This study had some limitations. First, harvested hamstring muscles were used to examine the slope of increase in shear modulus of individual muscles. Although the BFlh and ST share a common proximal tendon [19, 20], the intermuscular connections were transected in this experiment to assess individual muscle elongation. Furthermore, the BFlh and BF short head were also not connected distally in this study. Considering that the shear modulus decreased after intermuscular connection dissection in our latest study [30], neighbouring muscles may have influenced the shear modulus. In the future, it will be necessary to clarify the effect of intermuscular connections in the hamstring muscles on the elasticity of the muscles. Second, the application of repetitive loading may have altered tissue mechanical properties during the experiments. However, as the experiments were conducted at sufficient intervals and the test–retest reliability for the shear modulus was excellent at each site for all muscles, the effect on the main results was estimated to be minimal. Third, all cadavers were from elderly individuals. A previous study using SWE showed that the muscles of the elderly had a lower shear modulus than those of younger individuals [31]. However, differences in the association between the passive range of motion and tissue stiffness between younger and older adults showed an increase in the shear modulus with joint angle change regardless of age [31]. Therefore, the effect of aging on the shear modulus–passive force relationship obtained in this study was estimated to be small. Fourth, the mechanical properties of Thiel-embalmed cadavers could be different from those of living humans. However, previous studies have reported that the stiffness of Thiel-embalmed cadavers was verified against historical human data, and the stiffness of the cadavers measured using SWE was comparable in quality to that of living individuals [32]. These results indicate that SWE is a promising tool to evaluate the mechanical properties of Thiel-embalmed cadavers. In addition, the response to tissue elongation was found to be nearly identical for different fixation methods [23]. Therefore, the effect of fixation methods on the relationship between shear modulus and passive muscle force obtained in this study was estimated to be small.

## Conclusion

In conclusion, our study showed that shear modulus was strongly correlated with passive muscle force in individual hamstring muscles at each site. Moreover, after normalization by muscle length and ACSA, there was no significant difference in the slope of increase in shear modulus among the muscles. This finding suggests that the local mechanical properties of individual hamstring muscles can be indirectly estimated using SWE, and the slope of increase in shear modulus reflects characteristics of the muscle architecture.

## Data Availability

The datasets used and/or analyzed in this study are available from the corresponding author upon reasonable request.
