# Correction: Relationship between shear elastic modulus and passive muscle force in human hamstring muscles using a Thiel soft-embalmed cadaver

**DOI:** 10.1007/s10396-024-01436-w

**Published:** 2024-03-20

**Authors:** Gakuto Nakao, Taiki Kodesho, Takuya Kato, Yu Yokoyama, Yuhei Saito, Yuki Ohsaki, Kota Watanabe, Masaki Katayose, Keigo Taniguchi

**Affiliations:** 1 Graduate School of Health Sciences, Sapporo Medical University, Sapporo, Japan; 2 Professional Post-Secondary Course (Physical Therapist), Sapporo Medical Technology, Welfare and Dentistry Professional Training College of Nishino Gakuen School Foundation, Sapporo, Japan; 3 Department of Rehabilitation, Hitsujigaoka Hospital, Sapporo, Japan; 4 Department of Rehabilitation, Heiseikai Hospital, Sapporo, Japan; 5 First Division of Anatomy, School of Medicine, Sapporo Medical University, Sapporo, Japan; 6 Department of Physical Therapy, School of Health Sciences, Sapporo Medical University, South-1, West-17, Chuo-Ku, Sapporo, Hokkaido 060-8556 Japan

**Correction: Journal of Medical Ultrasonics (2023) 50:275–283** 10.1007/s10396-023-01317-8

The article “Relationship between shear elastic modulus and passive muscle force in human hamstring muscles using a Thiel soft‑embalmed cadaver”, written by Gakuto Nakao, Taiki Kodesho, Takuya Kato, Yu Yokoyama, Yuhei Saito, Yuki Ohsaki, Kota Watanabe, Masaki Katayose and Keigo Taniguchi, was originally published Online First without Open Access. After publication in volume 50, issue 3, page 275–283 the author decided to opt for Open Choice and to make the article an Open Access publication. Therefore, the copyright of the article has been changed to © The Author(s) 2023 and the article is forthwith distributed under the terms of the Creative Commons Attribution 4.0 International License, which permits use, sharing, adaptation, distribution and reproduction in any medium or format, as long as you give appropriate credit to the original author(s) and the source, provide a link to the Creative Commons licence, and indicate if changes were made. The images or other third party material in this article are included in the article’s Creative Commons licence, unless indicated otherwise in a credit line to the material. If material is not included in the article’s Creative Commons licence and your intended use is not permitted by statutory regulation or exceeds the permitted use, you will need to obtain permission directly from the copyright holder. To view a copy of this licence, visit http://creativecommons.org/licenses/by/4.0.

The original article has been corrected.

